# Properties of Two-Variety Natural Luffa Sponge Columns as Potential Mattress Filling Materials

**DOI:** 10.3390/ma11040541

**Published:** 2018-03-31

**Authors:** Yuxia Chen, Kaiting Zhang, Fangcheng Yuan, Tingting Zhang, Beibei Weng, Shanshan Wu, Aiyue Huang, Na Su, Yong Guo

**Affiliations:** College of Forest and Garden, Anhui Agricultural University, Hefei 230036, China; sheherose@163.com (Y.C.); 15905690096@163.com (K.Z.); 18158860792@163.com (F.Y.); 18715068105@163.com (T.Z.); weng1942@163.com (B.W.); 18297556933@163.com (S.W.); huangaiyue1124@163.com (A.H.); suna122216@163.com (N.S.)

**Keywords:** luffa sponge columns, mattress filling materials, mechanical properties, water absorption and desorption, dimensional stability

## Abstract

Luffa sponge (LS) is a resourceful material with fibro-vascular reticulated structure and extremely high porosity, which make it a potential candidate for manufacturing light mattress. In this study, two types of LS columns, namely high-density (HD) and low-density (LD) columns, were investigated as materials for filling the mattress. The results showed that the compressive strength of HD LS columns was significantly greater than that of LD LS columns. However, the densification strains of the two types of LS column were both in the range of 0.6 to 0.7. Besides, HD LS columns separately pressed to the smooth plateau region and the initial densification region exhibited a partial recovery of instant height when they were unloaded, and then both of them showed no more than 4.2% of height recovery after being allowed to rest at a constant temperature and humidity for 24 h. In contrast, when LD LS columns were compressed to the smooth plateau region, the height recovery was less than 1.62% compared to when they were pressed to the initial densification region, and that was more than 15.62%. Similar to other plant fibers used as mattress fillers, the two types of LS columns also showed good water absorption capacity—both of them could absorb water from as much as 2.07 to 3.45 times their own weight. At the same time, the two types of LS columns also showed good water desorption. The water desorption ratio of HD and LD LS columns separately reached 76.86 and 91.44%, respectively, after being let rest at a constant temperature and humidity for 13 h.

## 1. Introduction

**Figure 1:**
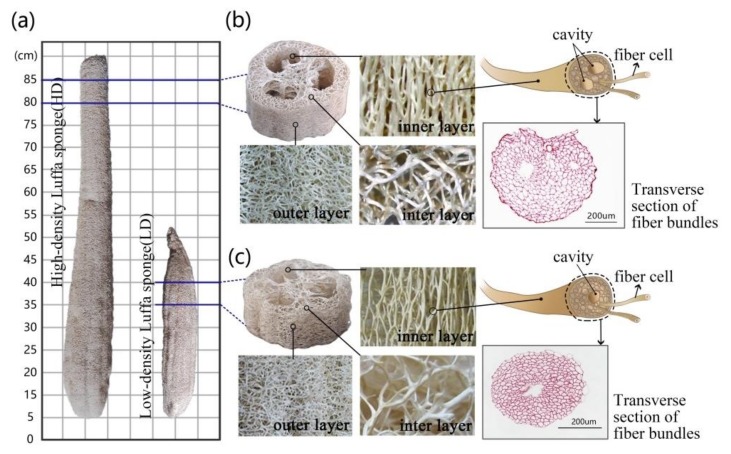
(**a**) high-/low-density luffa sponge; (**b**) high-density luffa sponges and their macro/micro structures; and (**c**) low-density luffa sponges and their macro/micro structures.

Mattresses made of natural plant fibers, such as jute, *Arenga engleri*, and other palms, are ecological, environmentally friendly, and bio-degradable, and they are extremely popular in the Chinese market. However, these mattresses have higher weights, higher firmnesses, and lower air permeabilities that lead to a reduction in comfort and transport convenience. Therefore, the search for a light-weight material with good air permeability as filling material for mattresses is essential. The luffa sponge (LS) commonly referred to as vegetable sponge or luffa cloth, is obtained from matured dried fruit of luffa cylindrica. Luffa has a fibro-vascular reticulated structure and is made up of an open network of random lattices of small cross-sections coupled with extremely high porosity (79–93%) and high specific pore volume (21–29 cm^3^/g) [1,2]. This indicates that LS has a potential to act as a promising candidate for manufacturing light-weight mattresses with good air permeability (4507 L/m^2^·s) [3]. Currently, LS is commonly used for dishwashing, bath sponge, cleaning automobiles, heat insulation, filters, packing materials, shock absorbers, and stuffing for pillows, saddles, shoulder pads, and shoe soles [1,4]. In addition, LS is also widely used in several other fields, such as pharmaceutical engineering [5,6], environmental engineering [7,8,9,10], biotechnology [11,12,13], and industrial products [14,15,16,17], and its good performance is attributed to its chemical stability. Furthermore, LS fiber bundles show good moisture absorption [1]. Related research has revealed that the human body emits 200 to 300 mL of body moisture each night, and 25% of this emitted moisture from the body surface is absorbed by the mattress during sleep [18]. This moisture should get transported to the environment in order to avoid a clammy feeling at the mattress surface, to avert mildew formation at the mattress bottom, and to prevent decubitus ulcers, because moist skin is rough and therefore more sensitive to shear forces. These mean moisture absorption and dissipation of mattresses significantly affect the quality of sleep. The previous research showed that the moisture content of high-density (HD) fibers ranges from 7.1% to 9.3%, while that of low-density (LD) LS ranged from 10.2% to 10.9%, and both of them were higher than that of jute and palm fibers [1].There are two common varieties of LS, namely, low density (LD) LS and high-density (HD) LS, as shown in Figure 1a. The HD LS, which belongs to the improved variety, is dense and thick. The thickness of the hoop wall is between 1.5 and 2.5 cm, the diameter of the fiber bundles is larger, and 2–3 holes are present in the cross-section of the fiber bundle [1,19], as shown in Figure 1b. LD LS has a thinner hoop wall with thin fiber bundles and a hole in the middle of the cross-section of the fiber bundle [1], as shown in Figure 1c. 

According to the structural features of LS, its structure is subdivided into the following four parts: outer surface, inner surface, middle layer, and inter layer [1,20]. The fiber bundles on the outer surface grow along the circumferential directions, while the fiber bundles on the inner surface grow along the longitudinal directions and have large diameters and straight texture. In the inter-layer between the inner surface and outer surface, the fiber bundles grow in all three directions, as shown in Figure 1b,c. In the middle region, the fiber bundles are along the radial direction and connect with the fiber bundles in the hoop wall [14]. In 2017, Chen et al. studied the structure and tensile properties of the two types of LS fiber bundle. They found that the diameters of fiber bundles, the thickness of cell wall, and the ratio of the wall to the lumen of HD LS were significantly larger than those of LD LS. However, the tensile strength, Young’s modulus (except fiber bundles in the middle region), and elongation at break of HD LS were lower than those of LD LS [1]. These results indicate that the microstructures and tensile properties of the two types of LS fiber bundles are very different. In 2012, Shen et al. studied the structural strength of HD LS. The results indicate that the density of LS column significantly affects its axial compression strength. Moreover, the axial compression curve of LS column exhibited the following three distinct stages: rapidly increasing elastic region, relatively smooth plateau collapse region, and densification region. The stiffness, compressive strength, and energy absorption capacity of HD LS columns are comparable to those of metallic foams, and it is a as good biomaterial for sustainable development [4,20,21]. In 2014, Chen et al. performed a multi-scale study to understand the relationship between the structural and mechanical properties at different levels of its hierarchical organization (the fiber of inner layer, foam-like blocks of lateral position, and different forms of columns) and different orientations, including the longitudinal direction, tangential direction, and radial direction. The results showed that the inner layer fibers of the hoop wall make significant contributions to the longitudinal properties of the LS column followed by the tangential properties, while the core part exhibits lesser mechanical strength than the inner layer [20]. However, the studies on LD LS columns have not been reported yet. Our previous studies showed that the LD LS fiber bundles exhibit good structural properties. In order to explore more potential applications of LS, extensive research effort is required to study of the mechanical properties of LD LS columns. Furthermore, due to the anisotropic and multi-hierarchical network structure of LS, whether and how to batch prepare LS mattress filling materials with homogeneous performance and dimensional stability need further study.

The main objective of this study was to investigate whether the hygroscopicity and moisture dissipation performance of these two types of LS as mattress filling materials meet the needs of healthy sleep. It is noteworthy that fluffy LS fibers are used in filling mattresses or pillows; however, the fluffy state destroys the natural network structure of LS, which leads to the collapse of mattress or pillows and makes them firmer after a while [22]. Therefore, a lot more systematic exploration is required to take full advantage of the natural network structure of LS as a filling material for mattresses. 

In this study, the two types of LS columns were used as the mattress filling materials by compressing them to a specified thickness. The compressive mechanical properties of LS columns, the dimensional stability of compressed LS columns, and the water absorption and desorption properties of LS as a mattress filling material were investigated by comparing with three-dimensional (3D) jute, palm, and jute mattresses.

## 2. Materials and Methods

### 2.1. Sample Preparation

**Figure 2:**
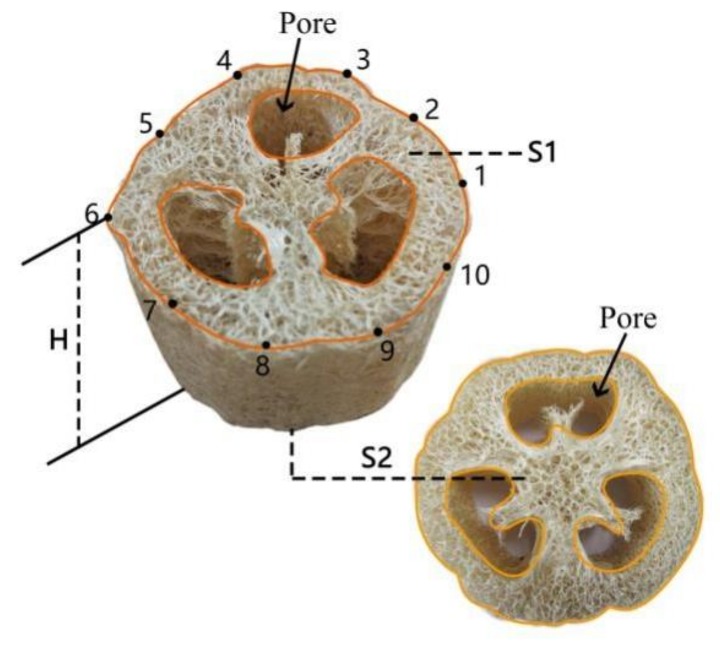
The method for testing the volume of luffa sponge column.

The two types of LS, i.e., HD (35–65 kg/m^3^) and LD (15–35 kg/m^3^) LS, used in our experiments were obtained from the Meier loofah Co., Ltd. (Jiangxi, China). Owing to aging contraction, the two ends of LS were amputated, and the central cylindrical part was selected for the experiment. First, the LS samples were cut into columns with heights of 50 mm, as shown in Figure 1b,c. Then, these columns were rinsed several times with water and dried. Subsequently, the LS columns were placed in a humidity chamber at 20 °C and 65% relative humidity (RH) for 24 h, in accordance with GB/T3903.33-2008. The density of LS columns was calculated by using Equations (1) and (2), as follows:

(1) ρ=m/v

(2) $$V=\frac{\left(S_{1}+S_{2}\right)\times H}{2}$$

where *ρ* is the density of a LS column (kg m^−3^), *m* is the mass of a LS column (kg), and *v* is the volume of a LS column (m^3^). *S*_1_ and *S*_2_ are the areas of the top and bottom transverse sections of a LS column (excluding the porous structure on the transverse section), respectively. The transverse section of an LS column and 10 observation points on the circumference are shown in Figure 2. The area is expressed in m^2^, while *H*, the height of LS column, is expressed in m.

HD and LD LS columns were randomly divided into the following three groups: the first group was used for the quasi-static uniaxial compression test and the unloading and reloading test; the second group was used for the water absorption and desorption test; and the third group was used for Fourier transform infrared (FTIR) spectroscopy analysis and morphological characterization with scanning electron microscopy (SEM).

The LS columns used for testing water absorption–desorption properties, were cut alongside the hoop wall, and the columns were flattened, and then trimmed into a 50 mm × 50 mm sheet. Finally, the sheets were stacked up into 50 mm thick samples. Furthermore, carvel jute, 3D jute, and palm mattresses were purchased from the market and cut into samples of similar sizes, and three duplicate specimens were obtained. The three plant fiber mattress were manufactured using hydrophilic low melting polyester fibers as adhesive to bind the dispersed plant fibers; the content of the low melting polyester fibers in carvel jute, 3D jute, and palm mattresses was 60%, 70% and 40%, respectively. The hydrophilic low melting polyester fibers were a composite of polyester and alkali-treating polyester fibers.

### 2.2. Morphology Observation of Luffa Sponge Fiber Bundles with Scanning Electron Microscopy

The surface and cross-sectional morphologies of the LS bundles before and after being hot-compressed were characterized by SEM (Hitachi S-4800 microscope, Tokyo, Japan). The SEM images were obtained at an accelerating voltage of 2 kV. The samples were sputter-coated with gold prior to observation [19].

### 2.3. Fourier Transform Infrared Spectroscopy Analysis

The Bruker Tensor II FTIR spectrophotometer was used to derive the FTIR spectra of the HD and LD LS fiber bundles. All the spectra were recorded in the wave number range of 500–4000 cm^−1^, operating in Attenuated Total Reflectance mode.

### 2.4. Quasi-Static Uniaxial Compression and Repetitive Loading Test of Luffa Sponge Column

The specimens of LS columns were placed in a humidity chamber at 20 °C and 65% RH for 24 h before performing the compression test. There were thirty duplicate specimens. Then, the compression performance of the LS columns was tested using a universal testing machine (Shimadzu Corporation, Shimadzu AG-X Plus, Kyoto, Japan) under ambient conditions at room temperature around (25 ± 2) °C with a RH of (50 ± 5)%. The crosshead speed was 3 mm min^−1^. The maximum compression of the LS columns was 70%. 

### 2.5. Dimensional Stability of Luffa Sponge Columns

The compressed LS columns (20 and 30 mm in height and 60 and 40% in compression) were placed in a humidity chamber at 20 °C and 65% RH for 24 h. The changes in the height of the LS columns were recorded before and after treatment under the constant temperature and humidity condition. The dimensional variation of LS column was calculated by using Equation (3) as follows:

(3) $$h=\frac{H_{2}-H_{1}}{50}\times 100\%$$

where *h* is the percentage of dimensional variation of the LS column, *H*_1_ and *H*_2_ are the heights (mm) of LS column before and after being subjected to a constant temperature of 20 °C and humidity of 65% for 24 h; both of them were the mean of 10 testing points, and five duplicate specimens were obtained.

### 2.6. Water Absorption and Desorption Testing

The water absorption and desorption of the four different samples (LS, 3D jute mat, palm mat, and jute mat) were tested in accordance with GB/T3903.33-2008. First, the samples with dimensions of 50 mm × 50 mm × 50 mm were placed in a humidity chamber at 20 °C and 65% RH for 24 h. Then the weight of samples was recorded as m_o_. Further, the samples were soaked in distilled water for 6 h; the samples were then taken out, drained until they had no dripping water, and the weight of samples was recorded as m_F_ at this time. After weighing, samples were placed in a constant temperature and humidity chamber, and the weight of samples was tested every 1 or 2 h and recorded as m_R_. The water absorption and desorption were calculated by using Equations (4) and (5), respectively, as represented below:

(4) $$C_{A}=\frac{m_{F}-m_{0}}{m_{0}}\times 100\%$$

(5) $$W_{D}=\frac{m_{F}-m_{R}}{m_{F}-m_{0}}\times 100\%\;$$

where *C_A_* represents the water absorption of sample (g/m^3^); *W_D_* represents the desorption rate of the sample (%); *m*_0_ is the original weight of the sample in a dry state (g); *m*_F_ is the final weight of the sample in a wet state (g); and *m*_R_ is the weight of the sample after environmental adjustment (g).

## 3. Results and Discussion

### 3.1. Basic Mechanical Properties of Two Types of Luffa Sponge Columns

**Figure 3:**
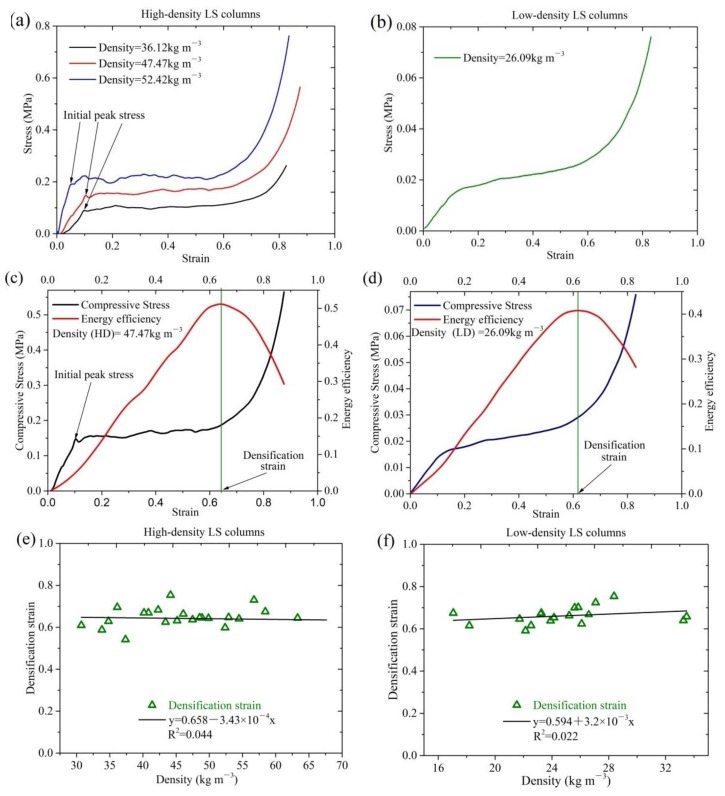
The mechanical properties of the two types of LS column: (**a**) the stress–strain curves of high-density LS columns, (**b**) the stress–strain curve of low-density LS columns; (**c**,**d**) illustration of the densification strain method; (**e**) densification strain of high-density LS columns; and (**f**) densification strain of low-density LS columns.

Figure 3a,b show that the compression progress of the HD and LD LS columns is similar to that of most other cellular materials. The deformation of the two types of LS column presents the following three different stages: an elastic stage, a plateau stage, and a densification stage. The obvious and smooth plateau stage indicates good toughness of LS columns [23]. The plateau stage curves of HD LS columns are approximate horizontal lines, as shown in Figure 3a, which indicates the good energy absorption capacity of HD LS columns [4]. However, the plateau stage curves of LD LS columns present a slight upward trend, as shown in Figure 3b. This indicates that LD LS columns have relatively good elasticity. Moreover, an initial stress peak in the stress–strain curves of the HD LS columns (Figure 3c) is also observed, while there is no peak stress in the case of the LD LS column (Figure 3d). 

Similar to other cellular materials, when the compressive strain of the LS column is small, the walls and struts of its cells are bent. If the stress on the walls and struts of the cells exceeds the initial peak stress of the LS columns, the plastic deformation appears in LS, and then the strain increases. However, the stress does not increase, and the static compression enters its plateau stage. In the entire process of deformation, LS fiber bundles first go through an elastic bending stage. When the stress exceeds the compressive strength of the LS fiber bundles, the fiber bundles on the inner surface start to bend, and the cavities in the interlayer and middle layer start to collapse. During the collapsing of cavities, the stress undergoes little change, thus the stress–strain curve of static compression exhibits a yield platform stage [24]. The HD LS column has a thick interlayer (Figure 1b), which indicates the presence of numerous cavities in the hoop wall. When static compression enters the plateau stage, the cavities gradually collapse. A thick interlayer indicates a long and smooth plateau. The existence of a long platform stage indicates that HD LS columns have good energy absorption capacity. In contrast, the LD column has a thin interlayer (Figure 1c), which indicates that the bending of the fiber bundles on the inner surface plays an important role in the process of compression deformation. We reported in our previous studies that the tensile strength, elongation at break, and Young’s modulus of LD LS fiber bundles were significantly higher than those of HD LS fiber bundles [1]. This further indicated that the deformation resistance and toughness of LD LS fiber bundles were significantly better than those of HD LS fiber bundles (Table 1). All these reasons are responsible for a slightly upward trend in the plateau stage of LD LS columns. However, HD LS columns have an initial stress peak and a relatively small strain corresponding to elastic deformation. In addition, the elastic stage strain is in the range of 0 to 0.1, the platform stage strain is in the range of 0.1 to 0.65, and the densification stage strain is in the range of 0.65 to 1, which is similar to the results reported by Chen et al. [20]. The compressive strength reflects the collapse stress value. Figure 3c,d demonstrate that the compressive strength of HD LS columns is nearly 10 times that of LD LS columns.

The densification strain is defined as the strain responding to the maximum energy dissipation efficiency value (Figure 3c,d), by using the following formulas [25]:

(6) $$E_{d}\left(\varepsilon_{a}\right)=\frac{\int_{0}^{\varepsilon_{a}}\sigma \left(\varepsilon \right)d\varepsilon }{\sigma_{a}},\;\;0\leq \varepsilon_{a}\leq 1$$

(7) $$\frac{dE_{d}\left(\varepsilon_{a}\right)}{d\varepsilon }/\varepsilon_{a}=\varepsilon_{i}=0,\;0\leq \varepsilon_{i}\leq 1$$

where $E_{d}$ is the energy dissipation efficiency, $\varepsilon_{i}$ is the strain, and $\varepsilon_{d}$ is the densification strain. 

Figure 3e,f present the densification strain of the two types of LS columns and their fitting curves to density. Clearly, the densification strain range of the two types of LS columns is similar; both of them range from 0.6 to 0.7. For the two types of LS columns, both the fitting lines of densification strain to density are approximately horizontal, and the values of their Adjusted R-square are 0.044 and 0.022, respectively. These indicate the absence of a significant linear correlation between the densification strain of the two types of LS columns and their densities (Table 1). Therefore, both types of the LS columns could be batch made into mattress filling material by controlling the compression ratio of column height.

### 3.2. Fourier Transform Infrared Spectroscopy Analysis

**Figure 4:**
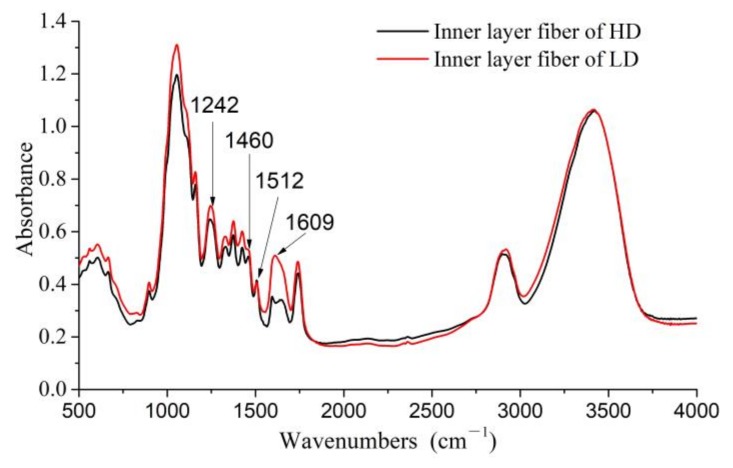
Fourier transform infrared (FTIR) spectra of high/low-density LS fibers.

Figure 4 shows the FTIR spectra of the fibers from the inner surface of the two types of LS columns. HD LS fiber exhibits well-defined bands at approximately 1460 cm^−1^. However, a well-defined absorbance peak is not observed at 1460 cm^−1^ in the spectra of LD LS fiber. The peak at 1460 cm^−1^ is associated with CH_2_ deformation stretching in lignin and xylan [26]. This indicates the existence of a methylene group in HD LS fibers. When the natural plant fiber is rich in methylene, the stiffness of its macromolecular chain is small, and the deformation resistance of the macromolecular chain reduces, which is beneficial for improving the flexural resilience of the fibers [27]. The absorbance peak centered at 1242 cm^−1^ is due to the C–O stretching vibration of the acetyl group [28]. Further, the peak at 1512 cm^−1^ is attributed to the C=C stretching of the benzene ring [29]. The peak centered at 1609 cm^−1^ indicates that the C=C aromatic stretching with a conjugated C–C bond [30]. All three peaks correspond to the characteristic bands of lignin. Figure 4 also demonstrates that LD LS fibers exhibit more explicit absorbance peaks centered at 1609 cm^−1^ than HD LS fibers. This indicates that LD LS fibers contain more lignin than HD LS fibers. More lignin is beneficial because it improves the stiffness of fibers. Our previous studies showed that the relative crystallinity, tensile strength, elongation at break, and elastic modulus of LD LS fiber bundles are higher than those of HD LS fiber bundles [1]. In contrast, lower crystallinity and more methylene are beneficial for increasing the flexural resilience of fibers [27]. 

### 3.3. Morphology Characteristics of Fiber Bundles from Compressed Luffa Sponge Columns

**Figure 5:**
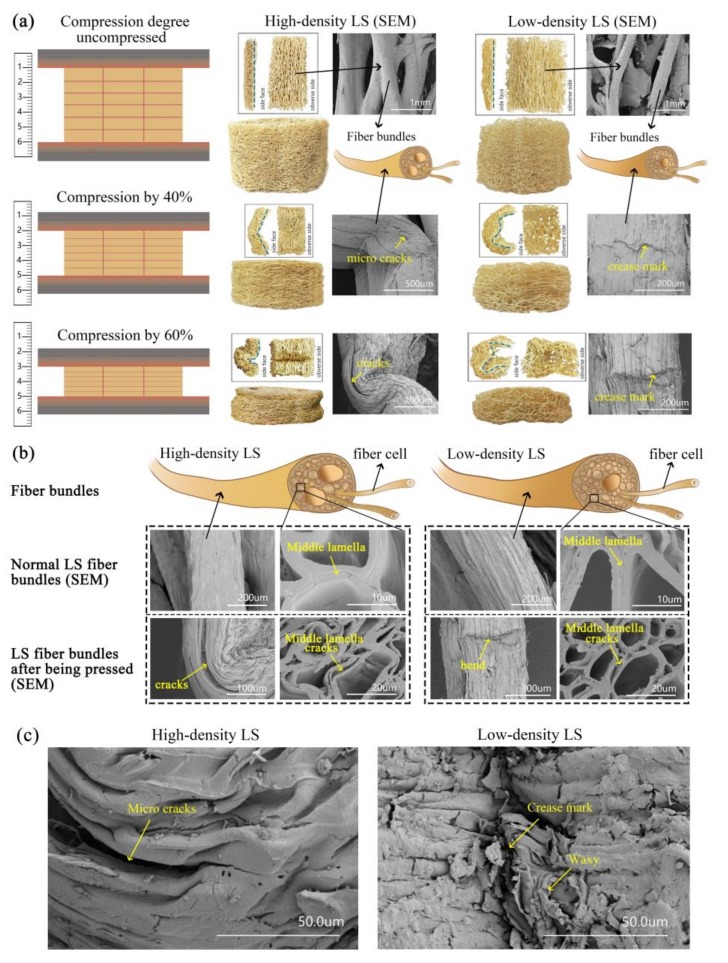
The morphology of the two types of LS columns and their fiber bundles: (**a**) the surface morphology of LS columns compressed to different degrees and the micro morphology of their inner fiber bundles, (**b**) the morphology of a fiber cell section before and after the compression of LS columns and (**c**) the surface morphology of HD and LD LS columns.

**Figure 6:**
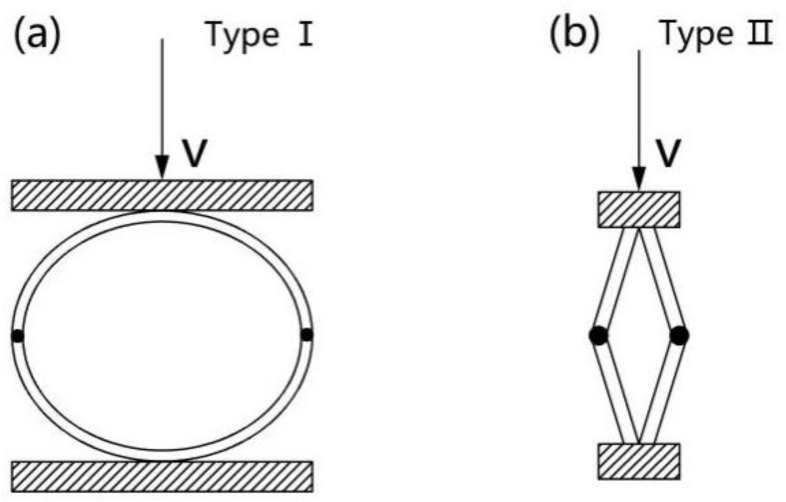
Two simple types of energy-absorbing structure.

Figure 5a demonstrates that the surface of the LS columns becomes even more compact and their hoop wall bends to some extent when they are compressed to the plateau stage. However, when compressed to the densification stage, the surface of the LS columns becomes more compact and their hoop wall shows the appearance of greater bends. A comparative analysis of the compression processes of the two types of LS columns indicates that for the HD LS column, the first bend occurs in the middle height part of the hoop wall, and then in the top and bottom parts of the middle bend. Owing to the thick hoop wall, the HD LS fiber bundles in the inside part of the bending center undergo severe bending collapses, while the fiber bundles in the outer part of the bending center undergo a relatively small degree of collapse. During the compression process, the hoop wall of the LD LS columns bends with soft curvature, while that of the HD LS columns bends with steep curvature. This shows that the two types of LS columns have different energy absorption structures. The bending deformation of HD LS columns is similar to half of type II structure [14], as shown in Figure 6b. Type II structure has obvious initial stress peak point and is an inertial sensitive structure [31]. However, LD LS columns are similar to half of type I structure, as shown in Figure 6a, and the mechanical properties of type I structure are less affected by loading speed.

The majority of the fiber bundles on the inner surface of the specimen are along the loading direction, and these fiber bundles contribute more to the larger enhancement in the compressive strength than those in the plateau stress region for the LS material [20]. Figure 5a shows the SEM images, exhibiting the appearance of microcracks on the surface (the lateral surface of the bending position) of the LS fiber bundles from the inner surface when the HD LS columns are compressed by 40%, and the microcracks develop into larger cracks when the HD LS columns are compressed by 60%. For LD LS fiber bundles, there are only crease marks on the inner surface, and no cracks occur on the bending position of the LS fiber bundles when LS columns are compressed by either 40% or 60%. This difference between the two types of LS fiber bundles may be closely related to the connecting structure between the fiber cells. Figure 5b demonstrates that the middle lamellas between HD LS fiber cells are thick, while those between LD LS fiber cells are thin. Middle lamellas are mainly composed of some pectin, which connect the LS fiber cells. Compared to fiber cell wall layers, middle lamellas are weak interfaces. Therefore, the ruptures occur first on the middle lamella when the fiber bundle is subjected to external force. When the HD and LD LS columns are compressed to some degree, their middle lamella ruptures to some extent, cracks occur between fiber cells, and fiber cells even separate from each other.

Although the macromolecular chain and fiber cells of HD LS bend easily, compared to LD LS, the fiber bundles of HD LS do not easily bend due to the fiber bundles’ relatively large diameters, low tensile strength, low elastic modulus, low elongation at break, thick middle lamella, and high proportion of substance [15]. Moreover, difficult bending of fiber bundles of HD LS leads to the debonding of fiber cells, and cracks occur in fiber bundles more easily when the LS columns are compressed. In contrast, the waxy layer on the surface of fiber bundles is crushed during the compression process of LD LS columns (Figure 5c), and there are only creases and no cracks at the bend position. 

### 3.4. Dimensional Stability of Compressed Luffa Sponge Columns

**Figure 7:**
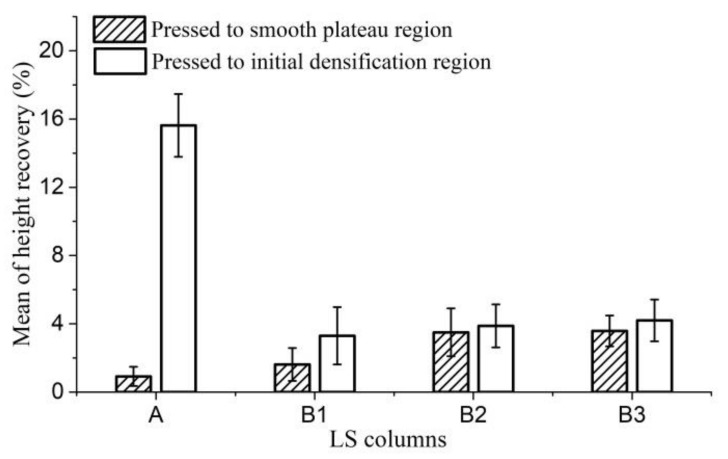
Mean of height recovery of LS columns. (A refers to LD LS columns with densities ranging from 15 to 35 kg/m^3^; B1, B2, and B3 refer to HD LS columns with densities ranging from 15 to 35 kg/m^3^, 41 to 50 kg/m^3^, and 50 to 65 kg/m^3^, respectively.

Figure 7 shows the height recovery of LS columns compressed by 40% and 60% after being set in a humidity chamber at 20 °C and 65% RH for 24 h. The flexural fiber bundle has a certain ability to recover the original shape under the action of internal stress, which is rebound resilience [27,32]. The shape recovery of plenty of LS fiber bundles leads to some degree of height recovery of compressed LS columns. The height recovery of LS columns compressed to the densification stage is larger than that of LS columns compressed to the plateau stage. This is attributed to the fact that the deformation of LS columns compressed to the plateau stage is mainly due to the collapse of the cavities, while that of LS columns compressed to the densification stage is mainly due to further deformation of fiber bundles. Lower density of LS columns leads to higher porosity. When the LS column with higher porosity is compressed to the plateau stage, its deformation is dominated by the collapse of cavities, not the elastic deformation of fiber bundles. Thus, when LS columns arecompressed to the plateau stage, their height recovery increases with density. 

Figure 7 shows that when the LS columns are compressed by 40% (to the plateau stage), the height recovery of the LD LS columns (15–30 kg/m^3^) is 0.9%, while that of B1 (HD LS columns of 30–41 kg/m^3^) is 1.62%, that of B2 (HD LS columns of 41–50 kg/m^3^) is 3.50%, and that of B3 (HD LS columns of 50–65 kg/m^3^) is 3.58%. When the HD LS columns are compressed by 60% (to the densification stage), the height recovery of B1, B2, and B3 are 3.30, 3.86, and 4.20%, respectively. Nonetheless, the height recovery of LD columns compressed by 60% is 15.62%. These results reveal the good dimensional stability of the compressed HD LS columns, and the height recovery in all cases is less than 5%. However, for LD LS columns, the amount of compression significantly affects the height recovery (Table 2 and Table 3). When compressed to the densification stage, the height recovery of compressed LS columns is great (15.62%), and these LS columns have poor dimensional stability. This is related to the mode of bending deformation (Type I) of the hoop wall of LD LS columns. This structure model has a stable support performance. Moreover, the higher tensile strength, larger elongation at break and elastic modulus of LD LS fiber bundles (that is LD LS fiber bundles have higher anti-deformation ability), lower substance in fiber cells, thin middle lamella in fiber cells, and larger porosity of LD LS fiber bundles [1], lead to the preservation of the intact surface morphology and inner structure of LD LS fiber bundles, when the fiber bundle undergoes a large bending deformation. The intact surface morphology and inner structure of fiber bundles is beneficial for the shape recovery of the bending fiber bundles, further contributing to the high height recovery of LD LS columns compressed to densification stage.

### 3.5. Water Absorption and Desorption Properties of Luffa Sponge

**Figure 8:**
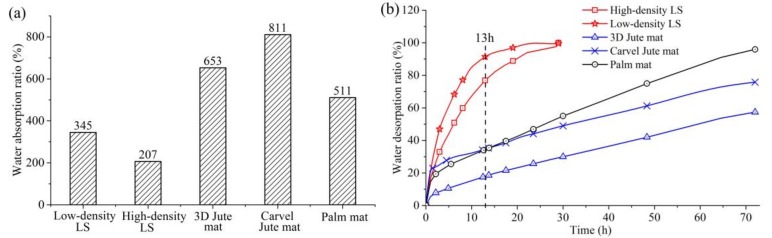
(a) Water absorption of five types of plant mattress filling materials, (b) water desorption of five types of plant mattress filling materials.

**Figure 9:**
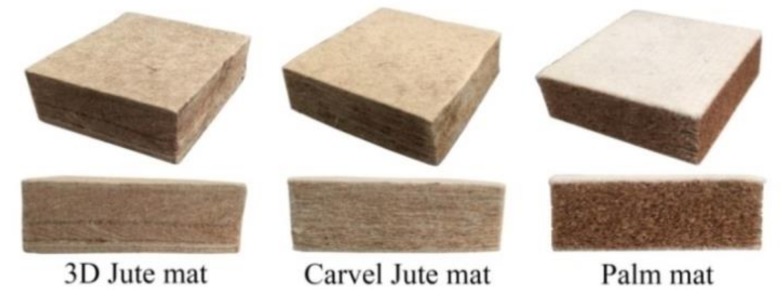
The three types of plant fiber mats.

Figure 8 and Table 4 show the water absorption and desorption properties of five plant fiber mattress filling materials (LD LS, HD LS, 3D jute mat, carvel jute mat, and palm mat). Figure 8a shows that the water absorption rate of LD LS (345%) is higher than that of HD LS (207%). This result is consistent with the results of our previous study on LS fiber bundles, which show that the moisture absorption of LD LS fiber bundles is higher than that of HD LS fiber bundles [1]. In addition, our previous studies showed that the moisture absorption of LS fiber bundles was significantly higher than those of jute fiber bundles and palm fiber bundles [1]. However, the water absorption of the two types of LS filling material is lower than those of 3D jute mats (653%), carvel jute mats (811%), and palm mats (511%). This should be related to the contents of hydrophilic low melting polyester fibers present in the three plant fiber mattresses. The three plant fiber mattresses were manufactured using hydrophilic low melting polyester fibers as adhesive to bind the dispersed plant fibers. These hydrophilic low melting polyester fibers are a composite of polyester and alkali-treating polyester fibers. The hydrophilic ability and water retention value of polyester fibers significantly increased after alkali treatment [33]. Figure 9 demonstrates that the content of hydrophilic low melting polyester fibers in carvel jute mats is maximal, followed by 3D jute mats and that of palm mats is the least. The water absorption tendency of these polyester fibers is higher than those of plant fibers. Thus, the water absorption of carvel jute mats is the highest, followed by 3D jute mats and palm mats. This shows that the contents of hydrophilic low melting polyester fibers significantly affect the water absorption of the three plant fiber mattresses.

Figure 8b, Table 4 and Table 5 demonstrate that the water desorption of the two types of LS filling materials is significantly higher than those of 3D jute mats, carvel jute mats, and palm mats. After 13 h, the LD LS filling material exhibited the highest water desorption rate of over 91.44 %, and the HD LS filling material also showed a higher water desorption rate of over 76.86%. Nonetheless, the water desorption rate of carvel jute mats and palm mats were only 35.61% and 35.77% respectively, and that of 3D jute mats was only 18.06%. In addition, it took 48 h and 72 h for palm mats and carvel mats, respectively, when their water desorption rates reached 75%, while the water desorption rate of 3D jute mat only reached 57% after 72 h.

A comparative analysis of the two types of LS filling material indicates that the water desorption of LD LS filling material is greater than that of HD LS, which is mainly due to the differences in the porosity of two types of LS and their structural characteristics [34]. LD LS is sparser and has higher porosity than HD LS. Therefore, the contact area of LD LS with air is larger than that of HD LS, and water desorption is faster than that from HD LS. 

The mattress dryness is crucial as, in a moist mattress, the surface of the cushion tends to produce a sticky feel, and the bottom is susceptible to mildew. To maintain the dryness of the human–bed interface, the LS mattress can dissipate the moisture emitted by the body during sleep to the surroundings much faster than the other three above mentioned plant fiber mattresses. 

## 4. Conclusions

In this study, two types of luffa sponge (LS) columns, namely, high-density (30–65 Kg/m^3^) and low-density (15–30 Kg/m^3^), were investigated as potential mattress filling materials. The following conclusions can be drawn:

(1) The stress–strain curves of high-density and low-density LS columns are similar; both of them have a long plateau stage. However, the high-density LS column has an obvious initial stress peak point in the elastic stage, and the energy absorption structure of its hoop wall belongs to the typical type II structure. The low-density LS column has no initial stress peak point in the elastic stage, and the energy absorption structure of its hoop wall belongs to type I. The compressive strength of the high-density LS columns is almost 10 times that of the low-density LS columns. However, the densification strain of the two types of LS columns is similar; both of them mainly range from 0.6 to 0.7. Therefore, the two types of LS columns can be batch-made into mattress filling material by grading the densities of LS columns. Moreover, the collapse of high-density and low-density LS columns is determined by the coupling of axial yielding and bending of the LS fibers.
(2) The high-density LS fibers contain relatively more methylene and a lower amount of lignin, which contributes to its good flexiblility and resilience to the macromolecule chain and microfibril of high-density LS fiber. The low-density LS fiber contains more lignin, which indicates that the microfibril structure of low-density LS fiber exhibits more rigidity. Even so, in the process of bending deformation of high-density LS fiber bundles, the debonding of fiber cells and surface cracking of fiber bundles occur easily due to the fiber bundles’ relatively large diameter, low tensile strength, low elastic modulus, low elongation at break, thick middle lamella, and high proportion of substance in fiber cells. However, the low-density LS fibers show more rigidity. There is also debonding between the fiber cells and crushing of the waxy layer on the surface of fiber bundles when the low-density LS columns are compressed to the initial densification stage; however, no cracks occur in the bent position—that is, fiber bundles of compressed low-density LS columns can maintain a relatively intact surface morphology.
(3) After compression treatment, high-density LS columns show good dimensional stability. Their height recovery is not more than 5% under constant temperature and humidity conditions for 24 h. However, the amount of compression significantly affects the dimensional stability of low-density LS columns. Low-density columns compressed by 40% (compressed to the platform stage) show a height recovery of no more than 1.62%, while those compressed by 60% (compressed to the initial densification stage) exhibit a height recovery up to 15.62%.
(4) Low-density and high-density LS filling materials can absorb 3.45 and 2.07 times their own weight of water, respectively. At the same time, the two types of LS filling material show good water desorption. The water desorption rates of low-density and high-density LS filling materials are over 91.44 and 76.86%, respectively, which are significantly greater than those of the other three commonly used plant fiber fillers for mattress. Therefore, the use of LS filling material to prepare mattresses is conducive to ensuring a dry bed during sleep.

## Figures and Tables

**Table 1:** Analysis of Variance (ANOVA) of compressive strength and densification strain of luffa sponge columns.

Factors	Types	Sum of Squares	df	Mean Square	F	Sig.
Compressive strength of luffa sponge (LS) columns (high-density–low-density; HD–LD)	Between Groups	0.224	1	0.224	77.580	0.000
	Within Groups	0.104	36	0.003	-	-
	Total	0.328	37		-	-
Densification strain of LS columns (HD–LD)	Between Groups	0.000	1	0.000	0.005	0.947
	Within Groups	0.053	36	0.001	-	-
	Total	0.053	37		-	-

**Table 2:** The statistical analysis of height recovery of LS columns.

LS Columns	The Amount Compressed	Mean of Height Recovery (%)	SD (*n* = 5)
A	40%	0.92	0.56
B1	40%	1.62	0.96
B2	40%	3.5	1.4
B3	40%	3.58	0.9
A	60%	15.62	1.84
B1	60%	3.3	1.68
B2	60%	3.88	1.26
B3	60%	4.2	1.22

Note: A refers to LD LS columns with densities ranging from 15 to 35 kg/m^3^; B1, B2, and B3 refer to HD LS columns with densities ranging from 15 to 35 kg/m^3^, 41 to 50 kg/m^3^, and 50 to 65 kg/m^3^, respectively.

**Table 3:** Analysis of Variance (ANOVA) of height recovery of LS columns.

Factors	Types	Sum of Squares	df	Mean Square	F	Sig.
HD LS columns compressed by 40% and 60%	Between Groups	5.985	1	5.985	3.177	0.086
	Within Groups	52.748	28	1.884	-	-
	Total	58.734	29	-	-	-
LD LS columns compressed by 40% and 60%	Between Groups	540.225	1	540.225	291.643	0.000
	Within Groups	14.819	8	1.852	-	-
	Total	555.044	9	-	-	-
HD and LD LS columns compressed by 40%	Between Groups	14.702	1	14.702	9.338	0.007
	Within Groups	28.338	18	1.574	-	-
	Total	43.039	19	-	-	-
HD and LD LS columns compressed by 60%	Between Groups	524.513	1	524.513	240.668	0.000
	Within Groups	39.229	18	2.179	-	-
	Total	563.742	19	-	-	-

**Table 4:** The statistical analysis of water absorption and desorption.

Materials	Water Absorption Ratio (%)		Water Desorption Ratio after 13h (%)	
	Mean Value	SD (*n* = 3)	Mean Value	SD (*n* = 3)
Low-Density LS	345	13	91.44	6.13
High-Density LS	207	4	76.86	4.56
3D Jute Mat	653	34	18.06	1.20
Carvel Jute Mat	811	6	35.61	1.19
Palm Mat	511	9	35.77	1.28

**Table 5:** Analysis of Variance (ANOVA) of water desorption of LS columns.

Factors	Types	Sum of Squares	df	Mean Square	F	Sig.
LS Columns and 3D Jute Mat	Between Groups	8736.66	1	8736.66	139.46	0.000
	Within Groups	438.51	7	62.64	-	-
	Total	9175.17	8	-	-	-
LS Columns and Carvel Jute Mat	Between Groups	4712.26	1	4712.26	75.23	0.000
	Within Groups	438.44	7	62.63	-	-
	Total	5150.70	8	-	-	-
LS Columns and Palm Mat	Between Groups	4681.25	1	4681.25	74.66	0.000
	Within Groups	438.88	7	62.70	-	-
	Total	5120.13	8	-	-	-
